# Supplementary material for: Predicting visual function by interpreting a neuronal wiring diagram
Source: Nature. 2024 Oct 2;634(8032):113–23. doi: 10.1038/s41586-024-07953-5 (PMC11446822; doi:10.1038/s41586-024-07953-5)
Supplement: Supplementary file 6 — CRF and ERF predictions for individual TmY4 and TmY9 cells. Analogous to Supplementary Data 3, but for TmY target types. Shown are the top four monosynaptic pathways, the strongest pathway passing through each of the top ten intermediary types (ranking from Extended Data Fig. 7), and the trisynaptic pathway Tm1–TmY–Dm3–TmY (see the section entitled Prediction of spatial normalization). [file 41586_2024_7953_MOESM6_ESM.zip › DataS4/TmY9qΓèÑ/720575940614443670.pdf]

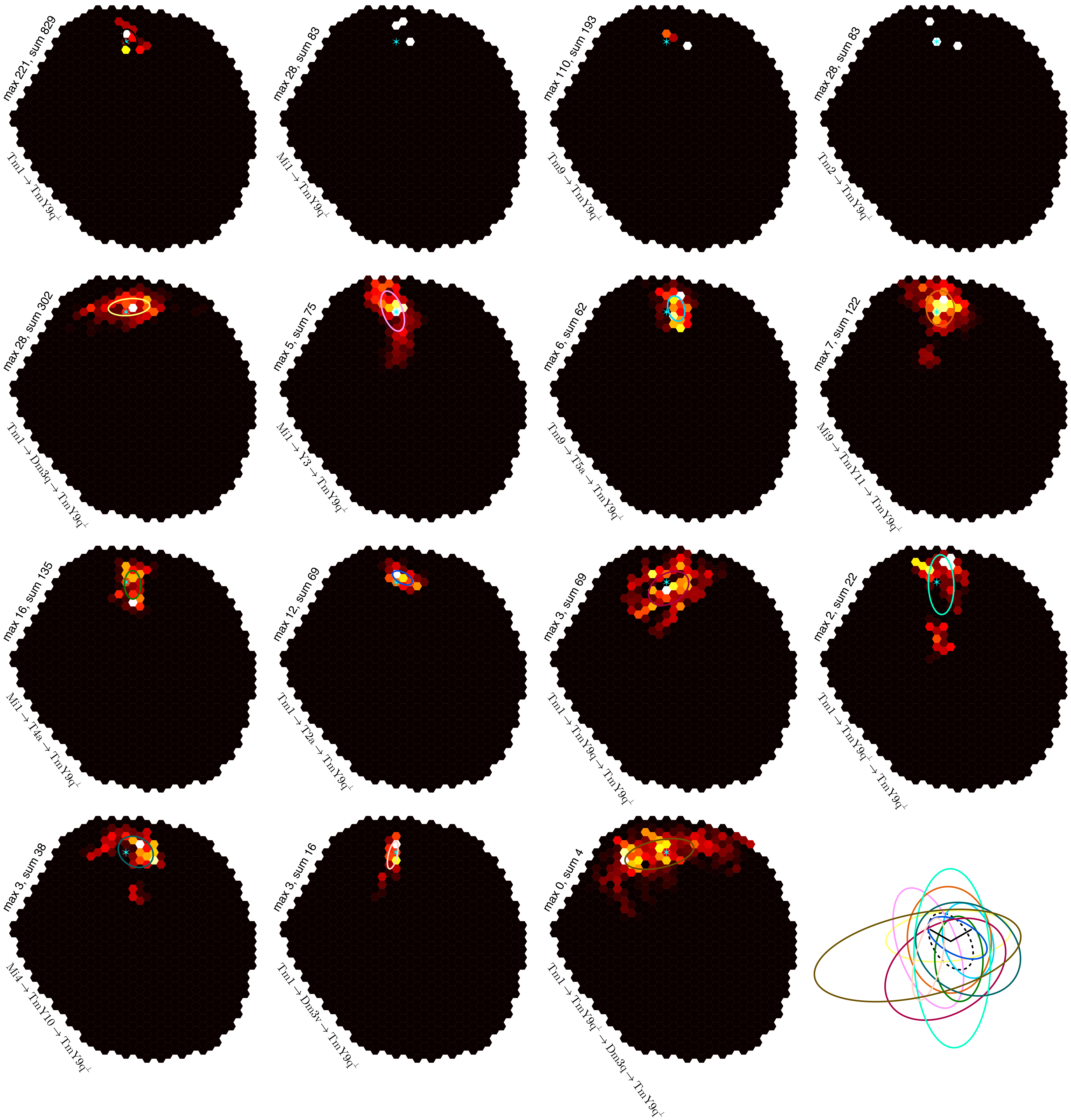

max 221, sum 829
Tm1 → TmY9q⊥
max 28, sum 83
Mi1 → TmY9q⊥
max 110, sum 193
Tm9 → TmY9q⊥
max 28, sum 83
Tm2 → TmY9q⊥
max 28, sum 302
Tm1 → Dm3q → TmY9q⊥
max 5, sum 75
Mi1 → Y3 → TmY9q⊥
max 6, sum 62
Tm9 → T5a → TmY9q⊥
max 7, sum 122
Mi9 → TmY11 → TmY9q⊥
max 16, sum 135
Mi1 → T4a → TmY9q⊥
max 12, sum 69
Tm1 → T2a → TmY9q⊥
max 3, sum 69
Tm1 → TmY9q → TmY9q⊥
max 2, sum 22
Tm1 → TmY9q⊥ → TmY9q⊥
max 3, sum 38
Mi4 → TmY10 → TmY9q⊥
max 3, sum 16
Tm1 → Dm3v → TmY9q⊥
max 0, sum 4
Tm1 → TmY9q⊥ → Dm3q → TmY9q⊥
